# Dissect the Dynamic Molecular Circuits of Cell Cycle Control through Network Evolution Model

**DOI:** 10.1155/2017/2954351

**Published:** 2017-03-30

**Authors:** Yang Peng, Paul Scott, Ruikang Tao, Hua Wang, Yan Wu, Guang Peng

**Affiliations:** ^1^Department of Clinical Cancer Prevention, The University of Texas MD Anderson Cancer Center, Houston, TX 77030, USA; ^2^Mathematical Sciences, Georgia Southern University, Statesboro, GA 30458, USA; ^3^University of California, Santa Cruz, CA 65064, USA; ^4^Department of Medical Oncology, Tongji Hospital, Tongji Medical College, The Huazhong University of Science and Technology, Wuhan, China

## Abstract

The molecular circuits of cell cycle control serve as a key hub to integrate from endogenous and environmental signals into a robust biological decision driving cell growth and division. Dysfunctional cell cycle control is highlighted in a wide spectrum of human cancers. More importantly the mainstay anticancer treatment such as radiation therapy and chemotherapy targets the hallmark of uncontrolled cell proliferation in cancer cells by causing DNA damage, cell cycle arrest, and cell death. Given the functional importance of cell cycle control, the regulatory mechanisms that drive the cell division have been extensively investigated in a huge number of studies by conventional single-gene approaches. However the complexity of cell cycle control renders a significant barrier to understand its function at a network level. In this study, we used mathematical modeling through modern graph theory and differential equation systems. We believe our network evolution model can help us understand the dynamic cell cycle control in tumor evolution and optimizing dosing schedules for radiation therapy and chemotherapy targeting cell cycle.

## 1. Introduction

**Figure 1:**
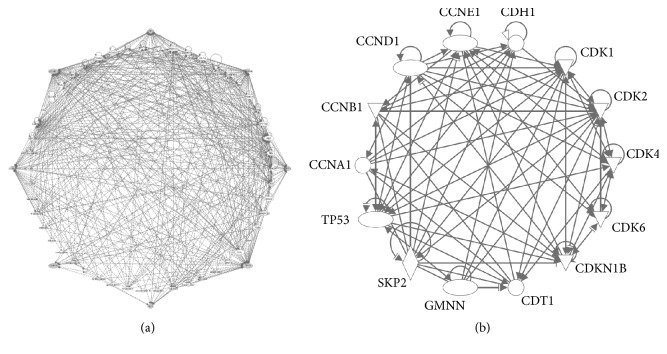
(a)* The complexity of cell cycle control*. IPA pathway analysis showed the regulatory protein network of cell cycle consists of a variety of biology signaling pathways and complex protein-protein interactions. (b)* A representative of protein-protein interaction network of cell cycle control*. Fourteen proteins involved in the cell cycle control were selected based on their key biological functions and relevance to human cancers.

Cell growth and division are regulated by molecular circuits known as “cell cycle control,” a coordinated protein-protein interacting network, that monitor cell proliferative signals, genome integrity, and proper timing of cell cycle transition from four different phases including S phase (DNA synthesis), M (mitosis), and two interphases (G1 and G2) between S and M phases [1]. A wide spectrum of biological pathways provides signaling inputs into molecular circuits of cell cycle control to determine how and when a single cell divides into two cells and also to ensure orderly cell cycle phase transition with high fidelity of cell duplication [1–3]. More specifically, the molecular circuits of cell cycle control are required by cells to respond to biological sensing systems including MAPK pathway, growth factor receptor pathways of EGFR, HER-2, and ErbB2-ErbB3, PI3K/AKT pathway, Wnt-*β*-catenin pathway, estrogen/androgen-mediated pathway, energy sensing pathway, metabolic pathway, and DNA damage response pathway (Figure 1(a)) [4–13]. Based on the signal inputs from these biological pathways, the molecular circuits of cell cycle control then generate decisive and robust signaling for cell growth. Thus, it is the key regulatory component to maintain cell homeostasis involved in a complex protein network (Figure 1(a)).

Given the functional importance of the molecular circuits of cell cycle control in integrating biological signals into cell growth decision, aberrant cell cycle control has been highlighted in the development of a variety of human diseases, particularly in human cancers, which contain a hallmark of uncontrolled cell proliferation [14–16]. For example, loss of a key cell cycle regulator p53 is found in more than 50% of human cancers [17, 18]. Overexpression of cyclin-dependent kinases (CDKs) and cyclin proteins (CCND1, CCNE1, and CCNB1) are found in many human cancers [19]. Overexpression of SKP2 which leads to reduced expression of negative cell cycle regulator CDKN1B is found in cancer cells as a bypass mechanism to escape cell cycle control [20].

The molecular circuits of cell cycle control are not only important for preventing the development of human cancers, but also important for determining treatment responses and toxicities to current chemotherapy and radiation therapy, most of which target cell proliferation and inhibit tumor growth [15, 21, 22]. For example, approximately 50% of cancer patients will receive radiation therapy, which induces DNA damage, arrests cell cycle progression, and leads to cell death. The efficacy of radiotherapy is largely affected by cell cycle control. Inhibition of cell cycle arrest signaling can leave cancer cells with less repair time and leads to a greater cell death to improve therapeutic responses [23]. The mainstay chemotherapeutic agents used in clinic, such as cyclophosphamide, cisplatin, 5-fluorouracil, gemcitabine, bleomycin, doxorubicin, etoposide, and topotecan/irinotecan, are targeting DNA as well [22]. They are also extremely toxic in normal tissues with the high proliferative rates such as epithelia of the gastrointestinal tract, hair follicle, and bone marrow. The selectivity of these agents between cancer and normal cells is largely determined by quantitative differences in the rates of cell division [22]. Thus, a better understanding of molecular circuits of cell cycle control will provide us with new insights into tumor evolution and anticancer treatments.

The regulatory mechanisms that control the cell cycle have been investigated in a huge number of studies. However, these studies often used conventional molecular biology approaches to dissect the function of each individual molecule. Because the molecular circuits of cell cycle control involve a variety of proteins and regulatory interactions, this biological complexity renders a great challenge in understanding the network impact of the cell cycle control by single-gene approaches. To address this challenge, a mathematical modeling of the molecular citrus of cell cycle control can be taken to simplify the complex biological circuits into a general framework for better analysis aimed at checking assumptions in addition to predicting.

Mathematical models correspond to conceptual representations that capture the essential features of the investigated process in the cell cycle and then omit details (i.e., elements that have negligible effects as well as elements that influence the explored behavior but are assumed as secondary properties) to describe its mechanisms. Mathematical models cast a process in the form of equations of a particular type to predict the system behavior and possibly suggest complementary experiments for a better understanding. The differential equations model is a continuous system in which the rates of change of the concentration of different states, such as genes, are related to the states in the state space either linearly or in a nonlinear setting [24]. Depending on the nature of the biological systems, different mathematical models based on ordinary differential equations were developed to study the progression of the system. Mamontov obtained sufficient conditions on nonautonomous ordinary differential equations that are capable of governing homeorhesis [25]. Dynamical modeling with differential equations has been shown to be effective in gaining insight of the cancer progression and response to the immunotherapy [26, 27]. The solution of the differential equations predicts the behavior of the biological system with much more details than the collected samples could reveal.

**Figure 2:**
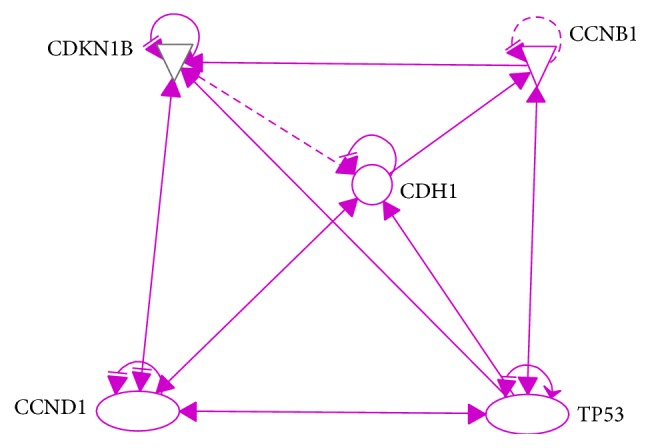
*A simplified network identified by IPA pathway*. Five key cell cycle regulators were selected to represent the complexity of protein-protein actions involved in the cell cycle regulation.

To establish molecular circuits of cell cycle control for mathematical modeling, we used QIAGEN's Ingenuity® Pathway Analysis (IPA®, QIAGEN Redwood City, https://www.qiagenbioinformatics.com//ingenuity) to generate a 14-protein network, which involve key proteins in regulating cell cycle and with extremely high relevance in human cancers including cyclin proteins (CCND1, CCNE1, and CCN), CDKs (CDK1, 2, 4, and 6), SKP2, CDKN1B, p53, and CDH1. GMNN and CDT1, two well established markers for G2/M phase and G1 phase, are included (Figure 1(b)). To further achieve simplicity of the core 14-protein network, we used IPA analyses to represent the protein-interaction network by 5 proteins including CDKN1B, CCNB1, CDH1, CCND1, and p53, which serve as molecular hubs for the circuits of cell cycle control (Figure 2).

## 2. Background and Methodology

The basic introduction to modern graph theory and random walks was described in our previous publication [28]. For completeness we include some of the details here. A graph consists of nodes and edges where each edge connects two nodes. Edges in a directed graph are directed, in the sense that each edge goes from one vertex to another but not necessarily vice versa. In our model, a directed graph is constructed such that every protein is represented by a node and every protein-protein interaction is represented by a directed edge between the nodes corresponding to the proteins. For instance, if protein *A* regulates protein *B*, there is a directed edge from the vertex corresponding to *A* to the one corresponding to *B*. In addition, we add two artificial nodes: an initial node (*S*) and a transition node (*T*). The node *S* has directed edges to and from all existing nodes. The node* T* has directed edges from all existing nodes and a directed edge to *S*.

In a random walk, a random walker starts from any chosen node. At each step, the walker moves along the directed edges to a neighboring node with equal probabilities. That is, if a node* A* has directed edges to *B*, *C*, and *D*, the random walker, when at *A*, will move to each of *B*, *C*, and *D* with probability 1/3. At each node, the directed edge from it to the transition node serves as the chance of exiting the current network to external proteins. Also, the directed edge from the initial node serves as the chance of restarting this random walk, representing the impact from external proteins outside of this network. Clearly, the higher the probability of a node being reached from other nodes is, the more interference the corresponding protein receives from other proteins.

Suppose the directed graph has *n* nodes, after *t* steps, *p*_*i*_ denotes the probability of the random walker being at the *i*th node. The vector *P*_*t*_ = (*p*_1_, *p*_2_, …, *p*_*n*_) is then the “state” after* t* steps. The sequence of *P*_*t*_ as *t* goes to infinity (i.e., the random walker keeps walking forever) forms a Markov chain. The states are also called the transition probabilities.

In order for such a Markov chain to converge, the corresponding graph must be “irreducible” [29] and “aperiodic” [30]. In simpler terms,

- the graph is “strongly connected”; that is, between every (ordered) pair of nodes there is a directed path;
- the greatest common divisor of all cycle lengths is 1.

We claim that both of these conditions are satisfied in our constructed network. First, the initial node *S* has a directed edge (hence, a directed path) to and from every other node in the graph. For any pair of nodes *A* and *B*, A → S → B → S → A  provides the necessary directed paths from* A* to* B* and vice versa. Thus, the graph under consideration is strongly connected. Second, for any node *A*, the directed cycle A → T → S → A  is of length 3. Also, for any directed edge *A* → *B*, the directed cycle A → B → T → S → A is of length 4. Therefore, the greatest common divisor of all cycle lengths is 1.

The unique limit to which this Markov chain converges is, in other words, the unique vector *P* to which the transition probability *P*_*t*_ converges as *t* approaches infinity. This limit *P* is the unique “stationary probability” or “stationary distribution.” Such a convergence indicates that if the random walking process goes on forever, the probability of each node (protein) being visited (i.e., being influenced through the network by other proteins) is a fixed value.

Using *M* to denote the adjacency matrix of the directed graph with edge weights corresponding to the probabilities (known as the transition matrix of this random walk), the stationary distribution can be directly determined by solving *PM* = *P*. In other words, an eigenvector corresponding to the eigenvalue 1, of the matrix *M* transposed.

We will use the vector *P* as a measure of how strongly each protein is performing in the network. As time changes, the variation of *P* with respect to each variable provides us with the necessary data to construct our differential equation model. We then use our model to predict the behavior of each protein in the network as well as the impact between the proteins.

First we construct our relation matrix from the 14-gene network in Figure 1(b), yielding Table 1 of their correlations, where an X in the *i*th row and *j*th column implies the fact that the* i*th gene regulates the *j*th gene, while Y denotes the binding relation between two genes. An entry labeled with X/Y simply means both regulation and binding relation exist between the two genes. We used the numerical labeling instead of gene names for concise presentation, while keeping the list of our labeling as in Table 2. With the addition of the transition node and restart node, we obtain a 16 by 16 adjacency matrix, from which our transition matrix is constructed as follows. Here we assume each outgoing edge from a chosen vertex is visited (by the random walker) with the same probability.

(1) $$\begin{array}{c} \left[\begin{array}{cccccccccccccccc} 0 & 0 & 0 & 0 & 0 & 0 & 0 & 0 & 0 & 0 & 0 & 0 & 0 & 0 & 0 & 1\\ \frac{1}{8} & 0 & 0 & 0 & 0 & \frac{1}{8} & \frac{1}{8} & \frac{1}{8} & \frac{1}{8} & \frac{1}{8} & 0 & 0 & 0 & 0 & \frac{1}{8} & \frac{1}{8}\\ \frac{1}{10} & 0 & 0 & 0 & \frac{1}{10} & \frac{1}{10} & \frac{1}{10} & \frac{1}{10} & \frac{1}{10} & \frac{1}{10} & 0 & 0 & \frac{1}{10} & 0 & \frac{1}{10} & \frac{1}{10}\\ \frac{1}{13} & \frac{1}{13} & 0 & \frac{1}{13} & \frac{1}{13} & \frac{1}{13} & 0 & \frac{1}{13} & \frac{1}{13} & \frac{1}{13} & \frac{1}{13} & \frac{1}{13} & \frac{1}{13} & 0 & \frac{1}{13} & \frac{1}{13}\\ \frac{1}{11} & 0 & \frac{1}{11} & 0 & \frac{1}{11} & 0 & 0 & \frac{1}{11} & \frac{1}{11} & \frac{1}{11} & \frac{1}{11} & \frac{1}{11} & \frac{1}{11} & 0 & \frac{1}{11} & \frac{1}{11}\\ \frac{1}{9} & \frac{1}{9} & \frac{1}{9} & 0 & 0 & \frac{1}{9} & 0 & 0 & 0 & \frac{1}{9} & 0 & 0 & \frac{1}{9} & \frac{1}{9} & \frac{1}{9} & \frac{1}{9}\\ \frac{1}{9} & \frac{1}{9} & \frac{1}{9} & 0 & \frac{1}{9} & 0 & 0 & 0 & \frac{1}{9} & \frac{1}{9} & 0 & \frac{1}{9} & 0 & 0 & \frac{1}{9} & \frac{1}{9}\\ \frac{1}{12} & \frac{1}{12} & \frac{1}{12} & \frac{1}{12} & \frac{1}{12} & \frac{1}{12} & 0 & 0 & \frac{1}{12} & \frac{1}{12} & \frac{1}{12} & \frac{1}{12} & \frac{1}{12} & 0 & 0 & \frac{1}{12}\\ \frac{1}{14} & \frac{1}{14} & \frac{1}{14} & \frac{1}{14} & \frac{1}{14} & 0 & \frac{1}{14} & \frac{1}{14} & \frac{1}{14} & \frac{1}{14} & \frac{1}{14} & 0 & \frac{1}{14} & \frac{1}{14} & \frac{1}{14} & \frac{1}{14}\\ \frac{1}{13} & 0 & \frac{1}{13} & \frac{1}{13} & \frac{1}{13} & \frac{1}{13} & \frac{1}{13} & \frac{1}{13} & 0 & \frac{1}{13} & \frac{1}{13} & \frac{1}{13} & \frac{1}{13} & \frac{1}{13} & 0 & \frac{1}{13}\\ \frac{1}{11} & 0 & 0 & \frac{1}{11} & \frac{1}{11} & 0 & \frac{1}{11} & \frac{1}{11} & \frac{1}{11} & \frac{1}{11} & \frac{1}{11} & \frac{1}{11} & \frac{1}{11} & 0 & 0 & \frac{1}{11}\\ \frac{1}{8} & 0 & 0 & \frac{1}{8} & \frac{1}{8} & 0 & 0 & \frac{1}{8} & 0 & \frac{1}{8} & \frac{1}{8} & \frac{1}{8} & 0 & 0 & 0 & \frac{1}{8}\\ \frac{1}{13} & 0 & \frac{1}{13} & \frac{1}{13} & \frac{1}{13} & \frac{1}{13} & \frac{1}{13} & \frac{1}{13} & \frac{1}{13} & \frac{1}{13} & \frac{1}{13} & 0 & 0 & \frac{1}{13} & \frac{1}{13} & \frac{1}{13}\\ \frac{1}{9} & 0 & 0 & 0 & 0 & \frac{1}{9} & \frac{1}{9} & 0 & \frac{1}{9} & \frac{1}{9} & 0 & 0 & \frac{1}{9} & \frac{1}{9} & \frac{1}{9} & \frac{1}{9}\\ \frac{1}{13} & \frac{1}{13} & \frac{1}{13} & \frac{1}{13} & \frac{1}{13} & \frac{1}{13} & \frac{1}{13} & \frac{1}{13} & \frac{1}{13} & \frac{1}{13} & 0 & 0 & 0 & \frac{1}{13} & \frac{1}{13} & \frac{1}{13}\\ 0 & \frac{1}{14} & \frac{1}{14} & \frac{1}{14} & \frac{1}{14} & \frac{1}{14} & \frac{1}{14} & \frac{1}{14} & \frac{1}{14} & \frac{1}{14} & \frac{1}{14} & \frac{1}{14} & \frac{1}{14} & \frac{1}{14} & \frac{1}{14} & 0 \end{array}\right] \end{array}$$

**Figure 3:**
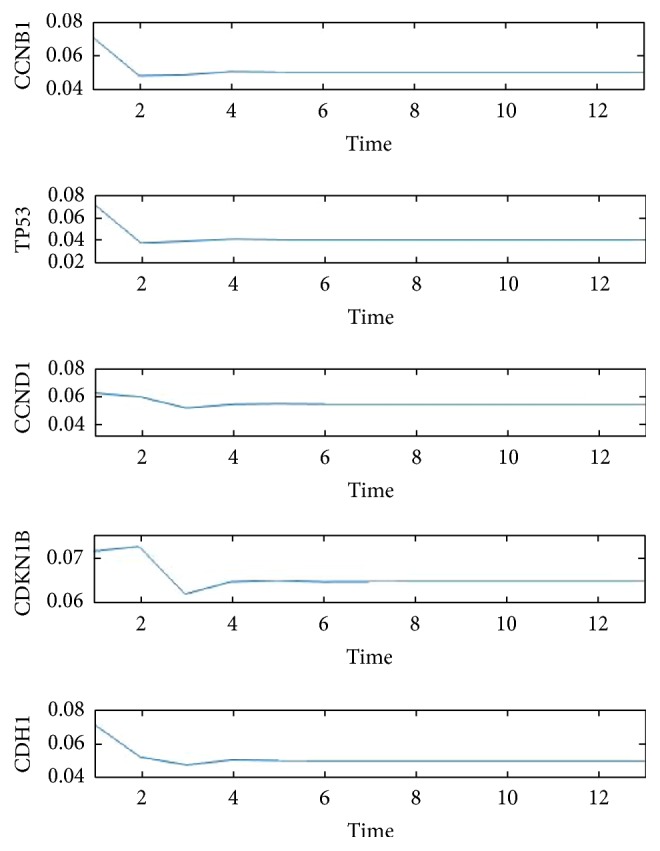
*Convergence of key gene expressions*. The expressions of the five key proteins CCNB1, CCND1, CDH1, CDKN1B, and TP53 are modeled through the random walk probability distribution, each converging to the stationary probability.

**Figure 4:**
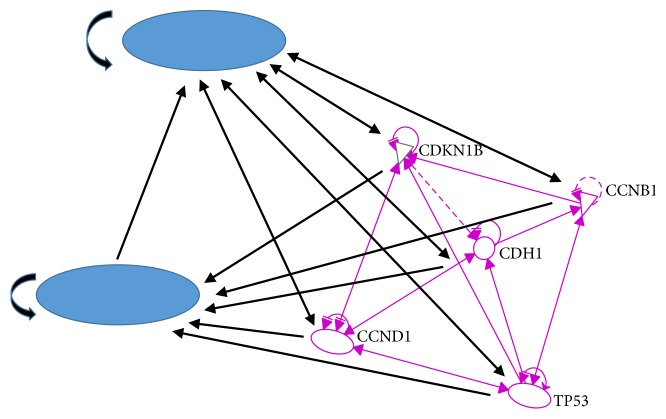
*Updated network with artificial nodes*. Two artificial nodes, the “transition” and “restarting” nodes, are added to the five-protein network to generate the directed graph for the random walk model.

Starting with a probability distribution (0, 1/14, …, 1/14, 0) (i.e., evenly distributed among the 14 genes), repeatedly applying the transition matrix provides us the sequence of data associated with each of the five key genes CCNB1, CCND1, CDH1, CDKN1B, and TP53, whose impact on each other is shown in Figure 2. We denote their expressions by the variables *x*_1_, *x*_2_, *x*_3_, *x*_4_, and *x*_5_, respectively. The convergence of each of these variables is shown in Figure 3. By projecting the differential of each key gene with respect to the neighboring genes (in Figure 2), we are able to model the evolution of these genes inside our original network through a system of differential equations, which enables us to predict and model the relations between individual pairs. The pseudo structure between them, together with the artificial transition and restarting points, is shown in Figure 4.

Based on the structure of the gene network and the collected data samples (i.e., the sequences of the expressions of each *x*_*i*_ when the hypothetical random walking process is applied), we propose the following system of differential equations that governs the evolution of the five-gene network:

(2) x˙1=r11x1+r12x2+r13x5,x˙2=r21x1+r22x2+r23x3+r24x1x3,x˙3=r31x2+r32x3+r33x4+r34x5,x˙4=r41x1+r42x3+r43x4+r44x5,x˙5=r51x2+r52x3+r53x4+r54x5,

where *x*_1_, *x*_2_, *x*_3_, *x*_4_, and *x*_5_ represent the genes CCNB1, TP53, CCND1, CDKN1B, and CDH1, respectively, in the gene network; *r*_*ij*_'s are the system parameters, controlling the rate of change of their corresponding states. These parameters are computed through calibrating the system with the sampling data of the state variables. The calibrating process includes a static stage followed by dynamic adaptation. In the static stage, the derivative samples are obtained from the data samples via a higher order finite difference method. The derivative samples along with the data samples are applied to (2) to optimally approximate the parameters by using the least squares method. This is carried out for each differential equation in (2). These newly computed parameters are applied to (2) for dynamic adaptation. At this stage, the dynamical system is simulated via the fourth-order Runge-Kutta method to produce the state trajectories. The state trajectories are plotted against the state samples for comparison. The discrepancies are reduced through transient-steady state compensation.

## 3. Results and Discussion

**Figure 5:**
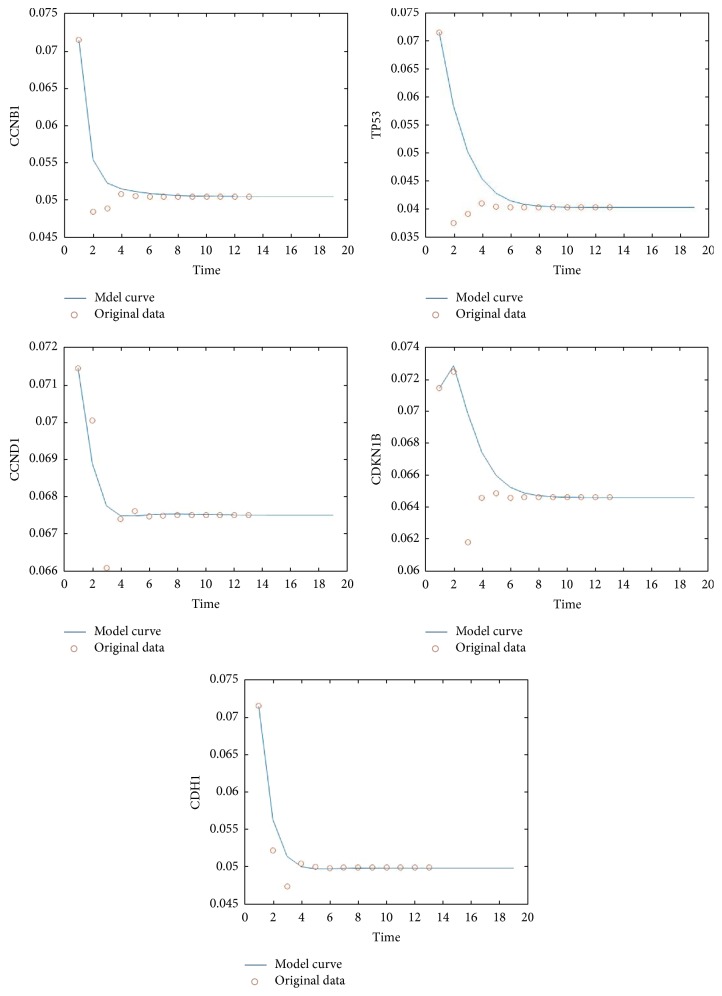
*Comparison between projected and sample data*. The state trajectories are plotted against the state samples for comparison. The discrepancies are reduced through transient-steady state compensation.

The simulation results of the differential equations system (2) against the collected sample data are shown in Figure 5. We compute the system parameters optimally by minimizing the errors throughout the transient and steady state stages. The numerical values of the parameters are listed in Table 3. Figure 5 shows the collection of state trajectories predicted by (2) along with the corresponding sample values. It is easy to see that the transient response and the steady state match up well with the collected samples.

In this study, we generate a mathematical model to predict dynamics of molecular circuits of cell cycle control. Instead of studying each molecules involved in cell cycle regulation, we use a network evolution model to dissect how molecular circuits of cell cycle control function as a network to maintain homeostasis of cell growth signals.

We believe our approach can be applied to a variety of biological contexts to solve key clinical questions in cancer research. First, cancer dormancy is a stage in tumorigenesis where the cells stop dividing but survive while waiting for appropriate endogenous and environmental signals to reenter into cell cycle and proliferate again [31]. Cancer dormancy is associated with drug resistance, tumor recurrence, and metastasis. Thus our network evolution model might provide a new perspective to identify the difference at the network level of cell cycle control between dormant cancer cells and proliferative cancer cells. The results from such analyses may mechanistically explain how dormant cells achieve withdrawal and reentry of cell cycle and more importantly may identify druggable targets that can be used to develop antidormancy therapy to extend patient survival.

Secondly, this network evolution model of cell cycle control might provide us with a molecular tool to monitor dynamics of cell cycle transition, which can be used to optimize dosing schedules of cancer preventive and therapeutic drugs. Most of radiation therapy and chemotherapy are given to cancer patients with scheduled cycles. For example, typical dosing schedule of radiation therapy is 2 Gy per day, 5 days per week, for 6 weeks [23]. However, it remains open as what alternative schedules could be applied to improve treatment efficacy and reduce toxicity. Our network evolution model provides us with a possible approach to model the treatment responses of cells to radiation by monitoring the dynamics of cell cycle control, which will help us guide the experimental validations to achieve an optimized dosing schedule.

In addition to the study of cancer treatment, our model may also be helpful for designing better cancer prevention regimens for patients with high risks of cancers. For example, estrogen signaling is a major biological pathway driving cell cycle progression and cell proliferation. For women at high risks of breast cancer such as genetic predisposition or existing premalignant pathological changes, tamoxifen and raloxifene have been demonstrated by clinical trials with approved chemopreventive effects [32, 33]. It has been recommended to take these drugs for 5 years in the cancer prevention setting based on clinical experience. However this prolonged dosing schedule causes severe side effects, which lead to reluctance of women at high risks of breast cancer to take these drugs for cancer prevention [32–34]. There is no method available to determine such an optimized dosing schedule with potential intermittent treatment in lieu of a 5-year continuous treatment. By applying our network evolution model, we may identify an optimal dosing schedule to conduct intermittent preventive treatment, which can reduce toxicity and improve efficacy [35]. Consistent with our findings, ordinary differential equation models have been widely used in cancer research to estimate tumor growth and anticancer treatment responses [35]. These studies demonstrate a proof of concept for using these models to simulate complex biological processes and interactions by developing simple quantitative models and also comparing experimental data.

In summary, we believe our interdisciplinary approaches may open a new avenue to study cell cycle control, which may better our understanding of tumor evolution and cancer prevention/therapy.

## Figures and Tables

**Table 1:** Correlations between the 14 genes.

	1	2	3	4	5	6	7	8	9	10	11	12	13	14
1	O	O	O	O	Y	Y	Y	Y	Y	O	O	O	O	Y
2	O	O	O	Y	Y	Y	X/Y	X/Y	Y	O	O	X	O	Y
3	X	O	X/Y	X	X	O	X/Y	Y	X/Y	X/Y	X/Y	X/Y	O	Y
4	O	X/Y	O	X	O	O	Y	X/Y	X/Y	X/Y	X/Y	X	O	Y
5	Y	Y	O	O	X/Y	O	O	O	Y	O	O	Y	Y	Y
6	Y	Y	O	Y	O	O	O	Y	Y	O	Y	O	O	Y
7	X/Y	Y	X/Y	X/Y	O	O	X/Y	X/Y	X/Y	X/Y	X/Y	O	O	Y
8	Y	X/Y	Y	Y	O	X/Y	X/Y	X/Y	X/Y	X/Y	O	X/Y	Y	Y
9	O	Y	X/Y	Y	Y	X/Y	X/Y	O	X/Y	X/Y	Y	O	Y	X/Y
10	O	O	Y	X/Y	O	X/Y	Y	X/Y	X/Y	X/Y	X/Y	Y	O	O
11	O	O	Y	Y	O	O	X/Y	O	X/Y	X/Y	X/Y	O	O	O
12	O	X	X/Y	X	X/Y	X	X	X/Y	X/Y	X/Y	O	X/Y	X	O
13	O	O	O	O	X/Y	X/Y	O	Y	Y	O	O	X	Y	Y
14	Y	Y	Y	X/Y	Y	X/Y	X/Y	Y	X/Y	O	O	O	Y	X/Y

**Table 2:** Numerical labelling of the 14 genes.

Numerical labeling	Gene names
1	CCNA1
2	CCNB1
3	CCND1
4	CCNE1
5	CDH1
6	CDT1
7	CDKN1B
8	CDK1
9	CDK2
10	CDK4
11	CDK6
12	TP53
13	GMNN
14	SKP2

**Table 3:** Determined system parameters.

*r*_11_	−1.6525
*r*_12_	0.56
*r*_13_	1.22
*r*_21_	0.21
*r*_22_	−1.72
*r*_23_	0.85
*r*_24_	0.3919
*r*_31_	0.32
*r*_32_	−1.65
*r*_33_	0.45
*r*_34_	0.38
*r*_41_	0.31
*r*_42_	0.23
*r*_43_	−1.62
*r*_44_	0.16
*r*_51_	0.24
*r*_52_	0.12
*r*_53_	0.11
*r*_54_	−1.9
